# Molecular characterization of invasive *Aedes albopictus* (Diptera: Culicidae) populations in northwest Iran: Insights into origin, insecticide resistance, and *Wolbachia* infection

**DOI:** 10.1186/s13071-025-07083-5

**Published:** 2025-10-29

**Authors:** Mohammad Bagher Ghavami, Nazanin Naseri Karimi, Mohammad Naghiloo

**Affiliations:** 1 Department of Medical Entomology and Vector Control, School of Medicine, Zanjan University of Medical Sciences, Zanjan, Iran; 2 Center for Management of Communicable Diseases, Deputy of Health, Zanjan University of Medical Sciences, Zanjan, Iran

**Keywords:** *Aedes albopictus*, Mitochondrial *COI* gene, *Vssc* gene, F1534L mutation, *Wolbachia w*AlbA and *w*AlbB superinfection, *Wolbachia w*AlbB strain

## Abstract

**Background:**

*Aedes albopictus*, a major invasive mosquito species and competent vector for dengue, chikungunya and Zika viruses, has recently become established in northern Iran. Developing effective control strategies requires elucidating the population structure, genetic origins, insecticide resistance mechanisms and prevalence of *Wolbachia* infection in these established populations.

**Methods:**

Eggs of *Aedes* species were collected in October 2024 using ovitraps deployed in high-risk areas of Tarom County, Zanjan Province, northwestern Iran. Egg specimens were reared to adulthood under standard laboratory conditions and subsequently subjected to molecular analyses, including sequencing of the mitochondrial cytochrome* c* oxidase subunit I gene (*COI*), screening for knockdown resistance (*kdr*) mutations in the voltage-sensitive sodium channel gene (*vssc*) and detecting *Wolbachia* infection via *Wolbachia* surface protein gene (*wsp*) amplification.

**Results:**

A total of 258 *Ae. albopictus* eggs were collected, from which 86 viable adults were reared. Mitochondrial *COI* gene analysis (1433-bp fragment) revealed 99–100% similarity to temperate-adapted Eurasian populations from China and southern Europe. The F1534L *kdr* mutation was detected in 45% of specimens (all heterozygous), indicating pre-existing pyrethroid resistance, while the remaining 55% retained the wild-type allele. Over 85% of samples were superinfected with *Wolbachia w*AlbA and *w*AlbB strains, and 12.5% harbored only the *w*AlbB strain.

**Conclusions:**

The establishment of a temperate-adapted *Ae. albopictus* population in Iran, coupled with a high prevalence of *Wolbachia w*AlbA and *w*AlbB superinfection strains and emerging insecticide resistance, underscores the urgent need for integrated vector management, continuous resistance monitoring and exploration of *Wolbachia*-based biocontrol strategies.

**Graphical Abstract:**

**Figure:**
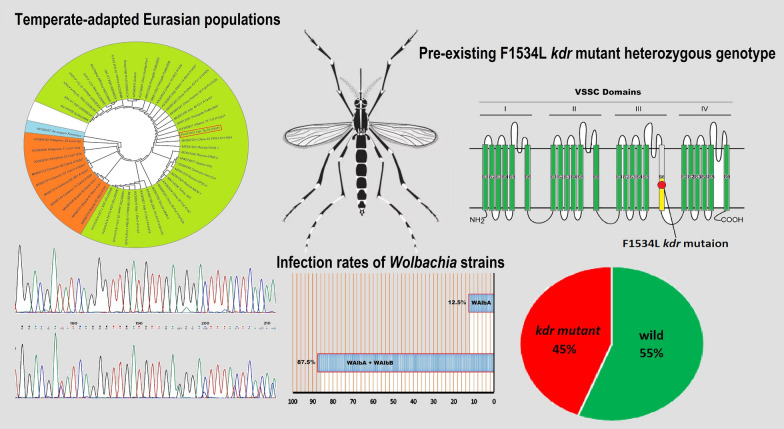

## Background

The Asian tiger mosquito, *Aedes albopictus* (Skuse, 1894) (Diptera: Culicidae), is a major global vector of arboviruses, including dengue, chikungunya and Zika viruses [1]. Recognized as one of the world's 100 most invasive species by the International Union for Conservation of Nature, it is considered among the most ecologically harmful invasive mosquitoes due to its high capacity for disease transmission [2]. Originally endemic to the tropical forests of Southeast Asia, *Ae. albopictus* has rapidly expanded its geographic range to nearly every continent in recent decades, facilitated by global trade, climate change and the species' remarkable physiological resilience and ecological adaptability [1, 3]. While climate change projections suggest potential range contractions in parts of Southeast Asia and the Americas due to extreme temperature thresholds, its prevalence has surged in the Middle East, with recent establishments reported in the South Caucasus, the western Caspian coast and northwestern Iran [4]. This rapid expansion highlights the urgent need for integrated vector management strategies to mitigate further proliferation and associated public health risks [5].

Accurate identification of invasive *Aedes* mosquitoes is critical for designing and implementing effective control strategies [6]. Although morphological and molecular characterization are primary identification methods, traditional taxonomic keys are often impractical due to their reliance on substantial expertise and inability to account for intraspecific genetic diversity. In contrast, DNA barcoding, particularly of the mitochondrial cytochrome* c* oxidase subunit I (*COI*) and nuclear internal transcribed spacer 2 (*ITS2*), provides precise, standardized species identification [7–9]. The maternally inherited *COI* gene, which undergoes minimal recombination, serves as a robust tool for assessing global genetic diversity and population structure in *Ae. albopictus*. Its rapid evolutionary rate in coding regions enables high-resolution discrimination of closely related lineages [10], thereby elucidating inter-population relationships and invasion pathways [11]. However, infection with the endosymbiont *Wolbachia* complicates mitochondrial DNA (mtDNA) analyses due to its maternal inheritance pattern and cytoplasmic incompatibility (CI) effects. These bacterial endosymbionts can induce mtDNA homogenization through selective sweeps, reducing host genetic diversity and rendering mitochondrial markers less reliable for reconstructing the evolutionary history of infected populations [12].

*Wolbachia* endosymbionts exhibit strain-specific variation in reproductive manipulation and pathogen-blocking phenotypes, making them promising tools for sustainable vector control strategies [13]. The *Wolbachia* surface protein gene (*wsp*) has been extensively used in phylogenetic studies of *Ae. albopictus* to characterize strains and their evolutionary relationships [14]. In *Ae. albopictus*, two predominant *Wolbachia* strains, *w*AlbA and *w*AlbB, are typically present. While superinfections (co-occurring *w*AlbA + *w*AlbB) are common in natural populations, males frequently harbor only *w*AlbB infection, suggesting sex-specific regulation of symbiont transmission or maintenance, potentially linked to host immune responses or tissue tropism [15]. *Wolbachia* prevalence is strongly influenced by climatic and geographic factors, with near-fixation of dual infections in stable tropical climates and strain-specific dominance (e.g. of *w*AlbB) in temperate regions due to thermal tolerance thresholds [16]. Altitude and urbanization further modulate infection patterns, with higher prevalence observed in lowland urban areas compared to rural or high-altitude sites, likely due to temperature-dependent transmission efficiency and host fitness trade-offs [17]. Understanding these ecological and evolutionary dynamics is crucial for optimizing *Wolbachia*-based biocontrol strategies, particularly given the impacts of climate change on tripartite symbiont–host–arbovirus interactions [18, 19].

Pyrethroids (PYs), a key insecticide class for *Ae. albopictus* control, face widespread resistance due to decades of overuse in agricultural and public health settings [20–23]. PYs target voltage-sensitive sodium channel (VSSC) proteins, and mutations in the *vssc* gene reduce pyrethroid binding, conferring knockdown resistance (*kdr*) [24, 25]. Key *kdr* mutations include substitutions in four domains: DI (V410L/A/G), DII (S989Y/P, S1000Y, I1011V, V1016G/I, T1520I), DIII (I1532T, F1534C/S/L/W/R) and DIV (D1763W/Y) [21, 26, 27]. Codon 1534 mutations (e.g. F1534C/S/L/W/R), which are widespread globally, are strongly associated with resistance to type I PYs (e.g. permethrin) [28–39]. Although the V1016I or T1520I loci alone confers minimal resistance, their synergistic combination with F1534 mutations results in high-level resistance to both type I and II PYs [22, 29, 32, 33, 40]. Conversely, the V410L mutation in the IS6 segment confers cross-resistance to both pyrethroid classes by altering channel gating kinetics [22, 41].

Resistance to PYs in *Ae. albopictus* was first reported in Asia during the late 1990s and early 2000s, driven by agricultural insecticide overuse and public health campaigns. Metabolic mechanisms (e.g. cytochrome P450 monooxygenases [P450s] and glutathione S-transferases [GSTs]) were found to mediate resistance to permethrin and deltamethrin [23, 42, 43]. By 2010, resistance had spread globally, facilitated by urbanization, international trade and the species' ecological adaptability. Key *kdr* loci (e.g. F1534 and V1016) in the *vssc* gene were later identified, revealing regional divergence: Southeast Asian strains predominantly rely on metabolic resistance (P450-mediated detoxification), whereas American and Mediterranean populations exhibit stronger *kdr*-linked resistance, likely due to distinct selection pressures from local insecticide regimens [21, 22, 26, 27, 32, 44].

In Iran, *Ae. albopictus* was first detected in Konarak County, southeastern Iran, in 2016 [45], but subsequent surveillance has not recorded its presence there in recent years. Instead, a significant northward range expansion has occurred, with the species establishing itself in northern Iran. Following its initial identification in Gilan Province in August 2023 [46], the mosquito has rapidly formed stable populations and expanded into neighboring regions. More recently, during targeted surveillance in September 2024, *Ae. albopictus* populations were confirmed in Tarom County, Zanjan Province, northwestern Iran, underscoring its continued spread. Understanding the molecular characteristics and prevalence of *Wolbachia* infections in these invasive populations is critical for developing targeted vector control strategies.

This study aimed to evaluate the *Wolbachia* infection status and strain types, assess population structure and genetic origin and determine pyrethroid resistance profiles in introduced populations of *Ae. albopictus* in Iran. Using molecular approaches, we analyzed the mitochondrial *COI* gene to clarify phylogenetic relationships and invasion pathways and screened the *vssc* gene for pyrethroid resistance-associated *kdr* mutations. By integrating *Wolbachia* infection data with genetic diversity and resistance profiles, our work enhances species identification accuracy, reconstructs dispersal patterns and informs region-specific control measures. These findings will guide tailored interventions—such as optimized insecticide deployment or *Wolbachia*-based biocontrol—to mitigate the global spread of this invasive species.

## Methods

### Mosquito egg collection and rearing

Mosquito eggs were collected in October 2024 from two locations in northwestern Iran: Ab Bar (36°55′48′′N, 48°56′39′′E) and Tashvir (36°48′47′′N, 48°58′12′′E). Sampling was conducted using ovitraps which consisted of black plastic 1-l buckets lined with light-brown Kraft paper strips. The strips were partially submerged in an attractant solution, prepared by mixing 100 ml of straw-infused water (made by soaking 50 g of straw and 1 g of baker's yeast in 1 l of water) with 400 ml of tap water to provide a moist substrate for oviposition by gravid females.

Ovitraps were strategically placed in fixed locations assessed to have high introduction potential and an elevated risk of contamination by invasive *Aedes* species. Traps were inspected weekly, and both the solution and paper strips were replaced. Egg-laden paper strips were examined for *Aedes* eggs using a stereomicroscope. Collected eggs were transported to the laboratory and reared in separate containers under standard insectary conditions (27 ± 2 °C, 75 ± 10% relative humidity and a 12:12 light:dark cycle). Adult *Ae. albopictus* specimens were identified morphologically [46] and stored at − 20 °C until DNA extraction.

### DNA extraction

Individual mosquitoes were homogenized in 500 µl of TENS lysis buffer (100 mM Tris–HCl [pH 8.0], 10 mM EDTA, 0.5 M NaCl, 1% [w/v] sodium dodecyl sulfate [SDS]). Proteinase K (20 µg) was added, followed by incubation at 55 °C for 3 h. Genomic DNA (gDNA) was extracted using a standard salt precipitation protocol [47], and the final pellet was resuspended in 30 µl of nuclease-free water. DNA concentration and purity were assessed using a NanoDrop 2000 spectrophotometer (Thermo Fisher Scientific, Waltham, MA, USA).

### PCR amplification, *kdr* genotyping and *Wolbachia* detection

Two mitochondrial *COI* gene fragments were amplified using universal primer pairs: 1490F (5'-GGTCAACAAATCATAAAGATATTGG-3') and 2160R (5'-TAAACTTCTGGATGACCAAAAAATCA-3') for the first fragment [48], and 2027F (5'-CCCGTATTAGCCGGAGCTAT-3') and 2886R (5'-ATGGGGAAAGAAGGAGTTCG-3') for the second fragment [49]. To detect *kdr* mutations, partial sequences of segment 6 within domains I–IV of the *vssc* gene were amplified. Primers used and their corresponding amplicon sizes for each domain are listed in Table 1. For *Wolbachia* detection, the *wsp* gene was amplified using primers wsp81F (5′-TGGTCCAATAAGTGATGAAGAAAC-3′) and wsp691R (5′-AAAAATTAAAACGCTACTCCA-3′), yielding a product of 590–622 bp [14].

**Table 1:** List of the primers used and the corresponding amplicon sizes for each domain of *Aedes albopictus* *vssc *gene

Domain	Primer name and sequences (5′to 3′)	Product size (bp)	References
I	Alb 410 F: GATAATCCAAATTACGGGTATAC AegScR10: GTGTTAGGATCAGGTGGACC	480	[This study, 26]
	Scf10: GTGTTACGATCAGCTGGACC AegScR10: (GTGTTAGGATCAGGTGGACC	160	[22, 27]
II	AegSCF20: GACAATGTGGATCGCTTCCC AegSCR20: GCAATCTGGGTTGTTAACTTG	472	[26, 27]
III	Alb171F: CCGATTCGCGAGACCAACAT AegSCR7: GACGACGAAATCGAAGAGGT	556	[This study, 26]
	AegSCF7: GAGAACTCGCCGATGAACTT AegSCR7: GACGACGAAATCGAAGAGGT	762	[26, 27]
IV	AlbSCF6: TCGAGAAGTACTTCGTGTCG AlbSCR8: AACAGCAGGATCATGCTCTG	278	[26, 27]

Each 25-µl PCR reaction volume contained 12.5 µl Red 2 × Master Mix (Amplicon, Odense M, Denmark), 8.5 µl ddH₂O, 1 µl (10 pM) of each forward and reverse primer and 2 µl (25–50 ng/µl) DNA template.

Amplification was performed in a SimpliAmp Thermal Cycler (Applied Biosystems, Thermo Fisher Scientific). The thermal cycling program for *COI* and *kdr* genes was an initial denaturation at 95 °C for 10 min, followed by 36 cycles of 95 °C for 30 s, 52 °C for 45 s and 72 °C for 45 s, with a final extension at 72 °C for 5 min. The protocol for the *wsp* gene was an initial denaturation at 94 °C for 5 min, followed by 30 cycles of 94 °C for 45 s, 50 °C for 30 s and 72 °C for 60 s, with a final extension at 72 °C for 5 min. PCR products were electrophoresed in a 1% agarose gel alongside a 1-kb DNA ladder (DM3100, SMOBIO, Seoul, Korea) for visualization.

Samples were considered to be *Wolbachia*-positive only if at least two independent PCR replicates were successful. Negative controls (nuclease-free water) were included to monitor for contamination. *Wolbachia*-infected *Drosophila melanogaster* specimens harboring the *w*Mel strain were used as positive controls.

### Data analysis

Amplicons were gel-purified and sequenced bidirectionally using ABI 3730xl technology (Applied Biosystems, Thermo Fisher Scientific). Sequences were assembled, aligned and compared against the GenBank database using National Center for Biotechnology Information (NCBI) BLAST (Basic Local Alignment Search Tool). BioEdit v7.7.1 (https://bioedit.software.informer.com/7.7/) was used for sequence alignment editing, and parsimony-informative sites were identified. Phylogenetic analysis was conducted in MEGA XII [50] using the neighbor-Joining method with 1000 bootstrap replicates (branch support threshold: ≥ 75%).

## Results

### Sample collection and DNA extraction

A total of 258 *Ae albopictus* eggs were collected from two sites: Tashvir (*n* = 150) and Ab Bar (*n* = 108). Under controlled laboratory conditions, 86 viable adult mosquitoes were successfully reared (49 from Tashvir and 37 from Ab Bar). High-quality genomic DNA was isolated from 64 adult specimens for downstream molecular analysis.

### Mitochondrial *COI* gene polymorphism analysis

Two *COI* gene fragments (707 bp and 839 bp, with a 113-bp overlap) were amplified and sequenced from 16 samples (8 per geographic region). Alignment produced a 1433-bp consensus sequence that was identical across all samples. BLAST analysis against 50 *Ae. albopictus COI* reference sequences from NCBI revealed 99–100% identity, indicating minimal genetic variation. The fragments exhibited a high A + T content (69.9%), with a base composition of 30% A, 39.9% T, 15.5% C and 14.6% G. Five *COI* sequences were deposited in GenBank under accession numbers PV017696, PV017745, PV017901, PV018825 and PV018826.

**Fig. 1:**
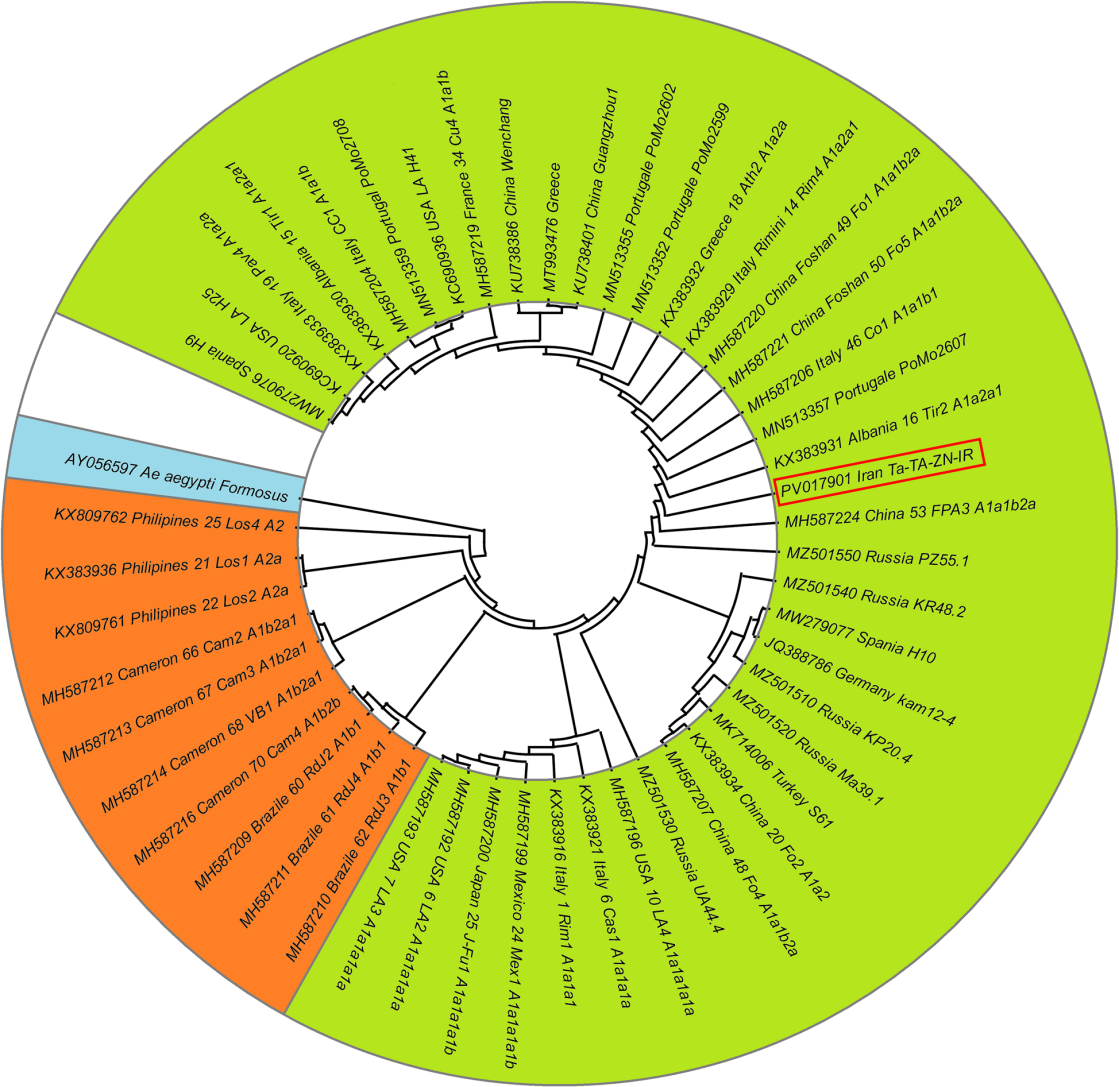
Phylogenetic tree of *Aedes albopictus* mitochondrial cytochrome* c* oxidase subunit I (*COI*) sequences constructed using the neighbor-Joining method. The analysis included sequences retrieved from NCBI GenBank, with *Aedes aegypti formosus* (accession AY056597) serving as the outgroup. The representative sequence from this study (PV017901) is highlighted by a red box

Phylogenetic analysis based on the *COI* genes revealed that the Iranian *Ae. albopictus* samples cluster closely with samples from Germany, Russia, Italy, and China, representing temperate Eurasian populations. In the phylogenetic tree, the Iranian samples grouped with specimens from China, southern Europe (Albania, Greece, Portugal, Spain) and North America, forming a distinct northern temperate clade. The sister group to this clade comprised samples from tropical regions, including Southeast Asia (Philippines), Africa (Cameroon) and South America (Brazil) (Fig. 1).

### Knockdown resistance mutation analysis

**Fig. 2:**
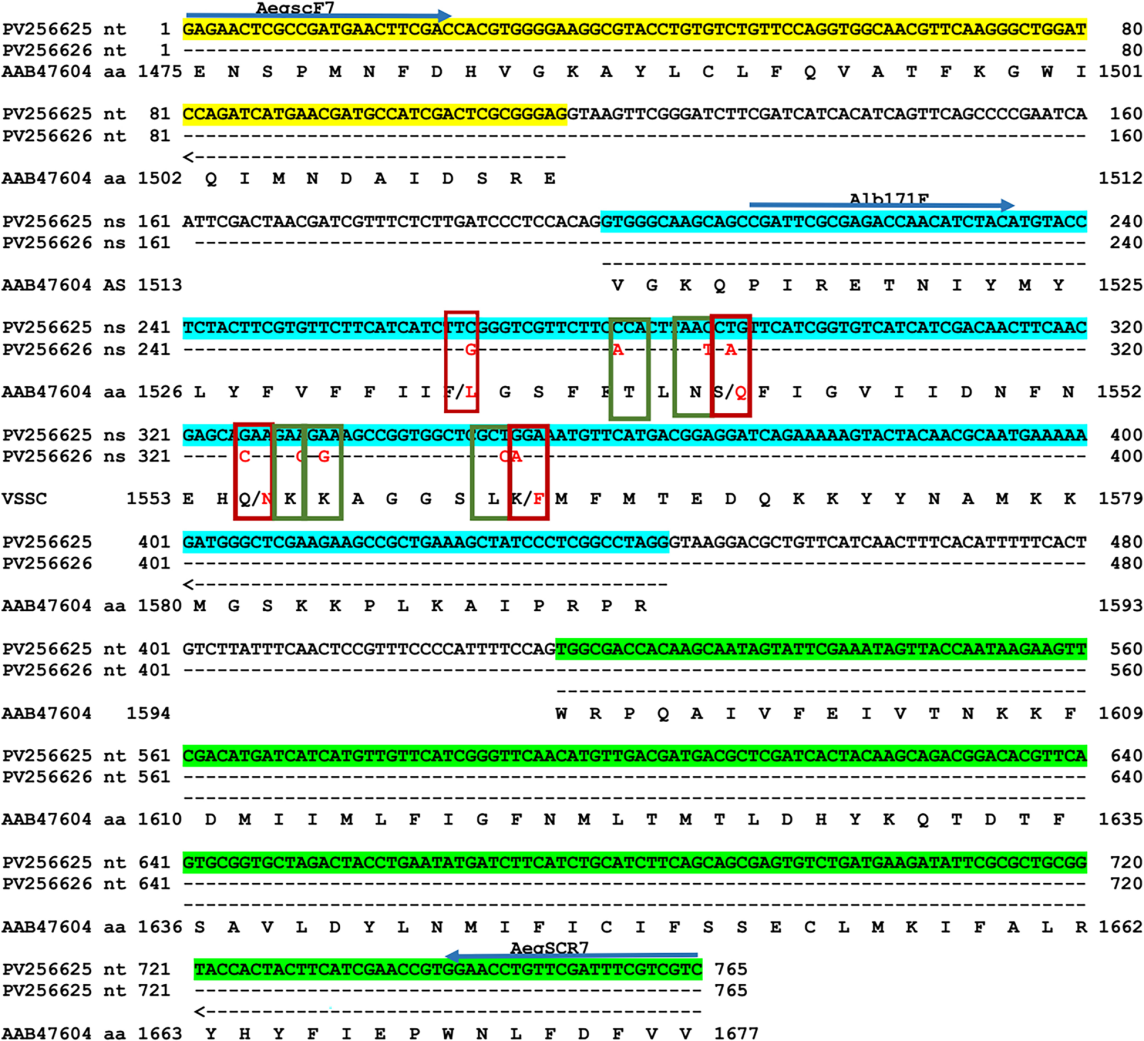
Sequence alignment of a fragment from domain IIIS6 of the voltage-gated sodium channel (*vssc*) gene, comparing a mutant *Aedes albopictus* strain (accession PV256626) with the corresponding wild-type allele (accession PV256625). Mutation sites are highlighted with vertical colored bars: red denotes missense mutations at amino acid positions F1534 (conferring knockdown resistance), S1542, Q1555 and K1562, while green indicates synonymous variants at T1539, N1541, K1556, K1557 and L1562. Gene structure annotations (nucleotide positions correspond to the aligned fragment) include the 5′ exon (nt 1–114, yellow), intron I (nt 115–197), a middle exon (nt 198–443, blue), intron II (nt 444–513) and the 3′ exon (nt 514–765, green). Deduced amino acid (*aa*) and nucleotide (*nt*) sequences are labeled adjacent to their respective regions. Primer-binding sites are marked with blue arrows. Residue numbering follows the *Musca domestica* sodium channel protein reference sequence (accession AAB47604)

Fragments of the *vssc* gene (domains I–IV) were amplified and sequenced from 42 individuals, achieving 80% coverage. Representative sequences were deposited in GenBank (accessions: PV032622, PV032624–PV032626, PV256625, PV256626). Amino acid numbering follows that of the house fly (*Musca domestica*) sodium channel protein reference (GenBank: AAB47604). No mutations were observed at key *kdr* loci (V410, L989, I1016, T1532, I1607). However, heterozygous parsimony-informative sites were detected in the domain III internal exon in 45% of samples (19/42). Within this exon, nine nucleotide substitutions were observed. One substitution, a C → G transversion at locus F1534, resulted in the F1534L *kdr* mutation. Five of the substitutions were synonymous (affecting the third codon position), while four were non-synonymous (involving the first or second codon positions) (Fig. 2).

### *Wolbachia* infection screening

**Fig. 3:**
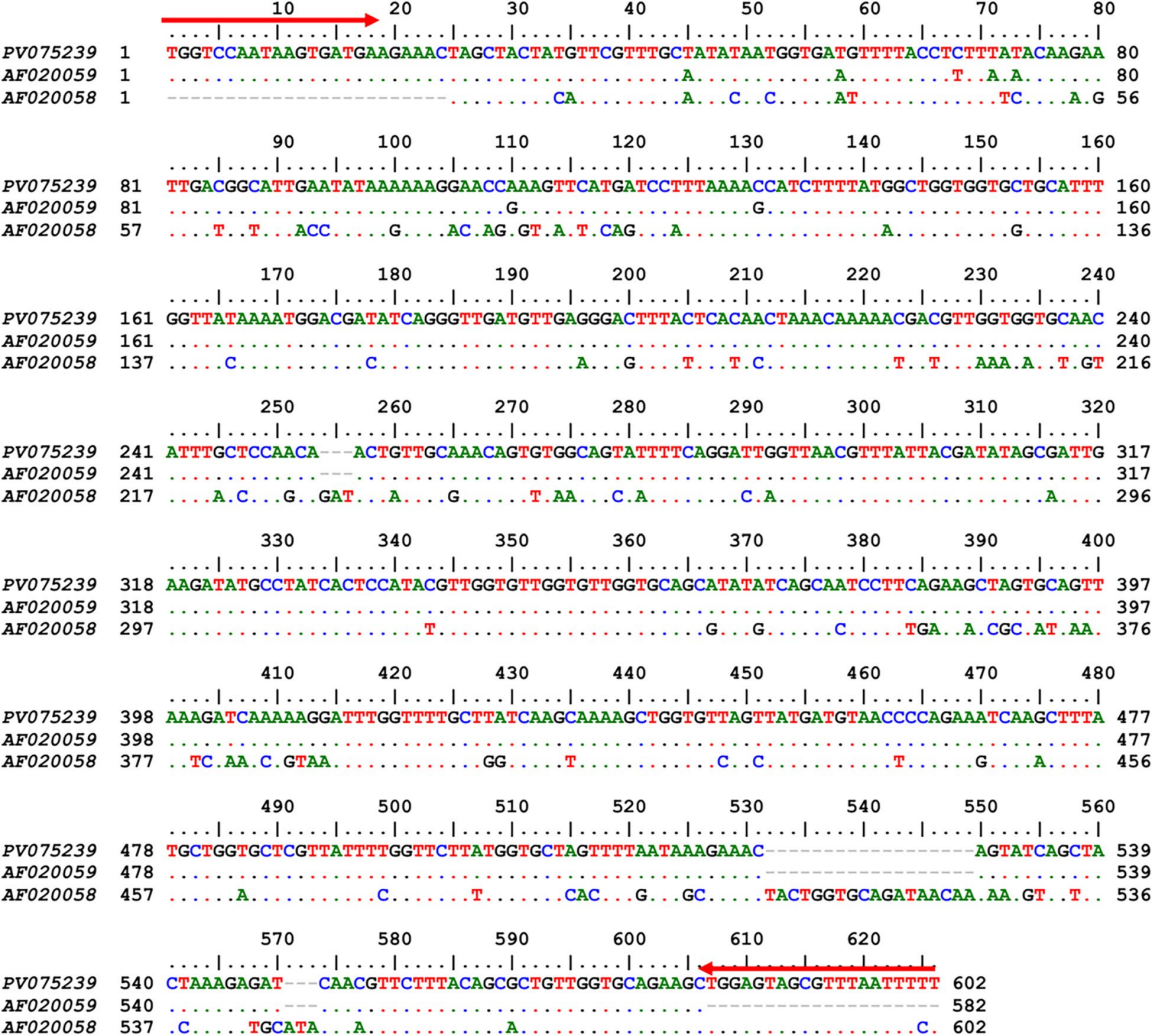
Multiple sequence alignment of *Wolbachia* outer surface protein (*wsp*) gene nucleotide sequences. The sequence obtained in this study (PV256626) is aligned against reference strains *w*AlbA (AF020058) and *w*AlbB (AF020059). Dots represent nucleotides identical to the reference sequences, dashes indicate deletions and red arrows mark primer-binding sites

**Fig. 4:**
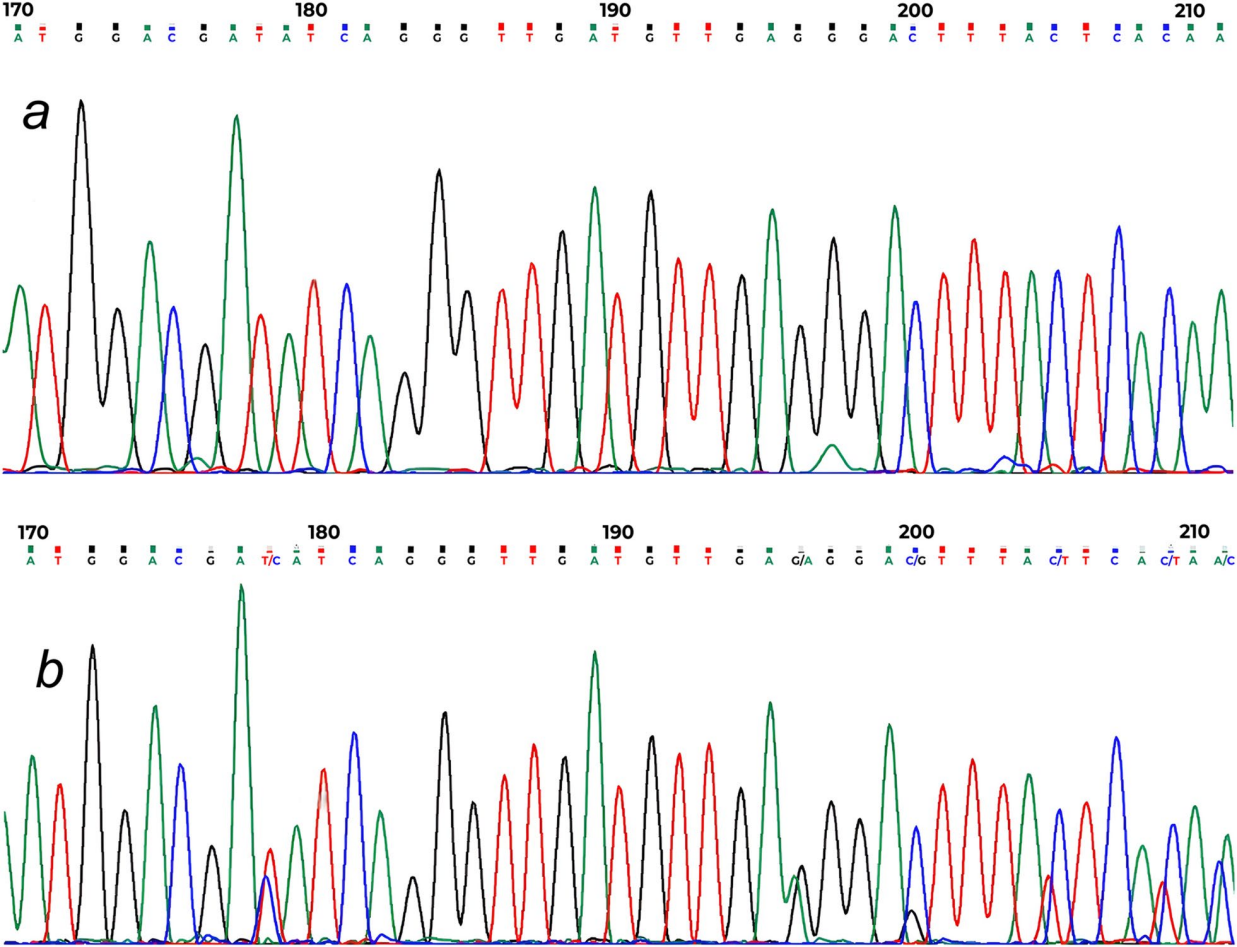
Chromatograms of the *Wolbachia wsp* gene fragment from infected *Aedes albopictus* mosquitoes in Iran. **a** Single infection with the *w*AlbB strain (supergroup B). **b** Mixed infection displaying overlapping peaks at nucleotide positions 178, 196, 200, 205, 209, and 211, which correspond to polymorphic sites between the *w*AlbB and *w*AlbA strains (supergroups B and A). This pattern indicates sequence heterogeneity consistent with a dual-strain co-infection

All 16 *Ae. albopictus* specimens screened from both sites tested positive for *Wolbachia* infection via PCR targeting the *wsp* gene, confirming a 100% infection rate. Sequencing identified two *Wolbachia* strains, *w*AlbA and *w*AlbB. Co-infection with both strains was observed in 87.5% (14/16) of the specimens. Single infection with only the *w*AlbB strain occurred in 12.5% (2/16) of specimens (Fig. 3, 4). A representative *w*AlbB sequence was deposited in GenBank (accession: PV075239).

## Discussion

The introduction and global spread of the Asian tiger mosquito pose significant public health concerns due to its role as a vector for arboviruses such as dengue and chikungunya [1, 2, 6, 10, 11, 51, 52]. Understanding the genetic origins of invasive populations is critical for assessing their ecological plasticity and adaptive potential under climate change [2, 53–57]. In this study, *Ae. albopictus* populations in northwestern Iran exhibited minimal genetic and phenotypic variation, suggesting constraints on local adaptation or a recent genetically homogeneous founding event.

MtDNA, particularly the *COI* gene, is a widely used molecular marker for tracing invasion pathways due to its maternal inheritance, high mutation rate and low recombination frequency [8]. This marker has been instrumental in phylogeographic studies of *Ae. albopictus*, elucidating population structures and dispersal patterns across diverse regions [11, 50, 58–61]. The reduced mtDNA diversity observed in the Iranian populations, which is consistent with previous findings in other invasive fronts [7, 39, 62], may reflect founder effects during colonization or *Wolbachia*-mediated selective sweeps. Although direct comparisons with native lineages were beyond this study's scope, our expanded *COI* dataset improves the resolution for reconstructing invasion routes into Iran and neighboring regions and offers valuable insights into the species' global invasion dynamics.

The high *COI* sequence homology between Iranian *Ae. albopictus* and populations from temperate regions (e.g. China, Italy, Germany, Russia) suggests that climatic bottleneck effects in these areas may have selected for cold-tolerant phenotypes pre-adapted to colonize northwestern Iran. This aligns with the *bridgehead effect*, where an invasive population in a secondary introduced range undergoes rapid adaptation, enhancing establishment and spread potential compared to its source population [34]. Such adaptive shifts could significantly amplify the risk of arboviral transmission in newly colonized regions, underscoring the critical need for proactive vector surveillance.

The heterozygous F1534L *kdr* mutation, which confers resistance to dichlorodiphenyltrichloroethane (DDT) and PYs like permethrin [24, 28], was identified in the *vssc* gene of Iranian *Ae. albopictus* mosquitoes. This mutation, which is associated with low-level pyrethroid resistance, has been documented in resistant populations across the Mediterranean region of southern Europe [27, 32] and China [32, 34, 35, 44, 61, 63, 64]. Given the close phylogenetic similarity of these Iranian populations to resistant populations abroad and the current lack of significant local insecticide pressure, the F1534L mutation was likely inherited rather than acquired through de novo adaptation. Its presence represents a serious public health concern, as pyrethroids remain critical for arbovirus vector control.

Of particular concern is the potential for the rapid expansion of insecticide resistance in Iran, driven by the unique breeding ecology of *Ae. albopictus*. This species thrives in water containers, discarded tires and other artificial receptacles in suburban and semi-urban zones. Consequently, larvae are chronically exposed to organic and chemical pollutants prevalent in these habitats. This persistent low-level exposure may create an environment that inadvertently selects for and accelerates the development of resistance. Although current pyrethroid resistance levels appear low, unregulated insecticide use or misguided interventions could rapidly accelerate the fixation of resistant alleles. The heterozygous status of F1534L indicates that resistance is not yet fixed, providing a crucial window for implementing targeted genomic and phenotypic surveillance. Proactive monitoring, especially in areas with high human-mosquito contact, is vital to track allele frequency dynamics, inform rational insecticide rotation strategies and mitigate resistance entrenchment. Prioritizing these measures now is essential to preserve pyrethroid efficacy and prevent irreversible resistance escalation.

High *Wolbachia* superinfection rates (*w*AlbA + *w*AlbB > 85%) were confirmed in Iranian *Ae. albopictus*. This result is consistent with recent studies from Manila, the Philippines (100%) [65]; Hainan, China (86.67%) [66]; the Yangtze River Basin, China (94.2%) [67]; eastern Thailand (100%) [68]; and Northeast Brazil (99.3%) [69]. The consistently high global prevalence of *Wolbachia* in this mosquito species strongly supports the conclusion that it is a natural host for these endosymbionts.

In the studied populations, infections with the *Wolbachia* strains *w*AlbA and *w*AlbB—including their co-infection (*w*AlbA/*w*AlbB)—were identified, a finding which is consistent with prior research [65–69]. The high frequency of dual infections suggests the stable vertical transmission of both strains, likely driven by robust maternal transmission fidelity in superinfected females, coupled with enhanced fitness traits such as increased egg viability, improved larval/pupal emergence rates, extended adult longevity and higher fecundity [15, 39, 70]. These synergistic advantages likely facilitate the species' invasion success in new ecosystems. Notably, *w*AlbB mono-infections have been found to predominate in regions with high dengue incidence, a pattern potentially attributed to this strain's elevated intracellular density and longer evolutionary history with *Ae. albopictus* [13, 18, 71]. This strain-specific prevalence could influence arboviral transmission dynamics, as *w*AlbB demonstrates a stronger pathogen-blocking capacity compared to *w*AlbA.

The high prevalence of *w*AlbA and *w*AlbB superinfections, coupled with their ability to enhance mosquito ecological fitness, underscores the necessity for sustained, integrated control strategies that move beyond reliance on insecticides. Approaches such as sterile insect techniques, *Wolbachia-*based population replacement and targeted habitat management are crucial to mitigate the public health risks posed by this invasive vector. Future research should prioritize biobanking local *Wolbachia* strains, quantifying the strength of CI in crosses between superinfected males and *w*AlbB-infected females and characterizing strain-specific density variations in both sexes to optimize field-based applications. These steps will be essential to refine *Wolbachia*-driven interventions and strengthen long-term vector control efforts.

Despite its valuable findings, this study has several limitations. First, potential sampling bias may have arisen from the exclusive use of ovitraps, which selectively attract gravid females. The incorporation of alternative methods, such as human landing catches [72] or BG-Sentinel traps [73], could improve the representativeness of future collections. Second, although the mitochondrial COI gene is effective for taxonomic identification, its maternal inheritance and low recombination rate limit its resolution for population genetic analyses. To gain a more comprehensive understanding of local population structure, future studies should integrate nuclear markers, such as odorant-binding protein genes [74] and hypervariable microsatellites [75]. Finally, the narrow focus on *kdr* mutation overlooks other critical resistance mechanisms. A complete characterization of resistance, including standardized bioassays (e.g. WHO tube tests) and multi-omics techniques (e.g. transcriptomics metabolomics) [76–78], is needed to elucidate both phenotypic resistance and its associated enzymatic/metabolic pathways.

## Conclusions

The results of this study confirm the presence of temperate-adapted *Ae. albopictus* populations in northwestern Iran, highlighting their successful establishment in the region. Proactive management strategies are urgently needed to limit their further spread and mitigate associated arboviral transmission risks. The detection of the heterozygous F1534L *kdr* mutation, linked to low-level pyrethroid resistance, underscores the critical need for ongoing resistance monitoring to inform rational insecticide deployment and delay resistance development. Future research should focus on conducting bioassays to quantify resistance frequency and intensity, employing multi-omics studies (genomics, transcriptomics, proteomics, metabolomics) to elucidate complex detoxification mechanisms and developing integrated vector management strategies with particular emphasis on elucidating the role of native *Wolbachia* strains in population dynamics for potential biocontrol applications. Given the high prevalence of *Wolbachia* superinfection and its potential to enhance mosquito fitness, sustainable, multi-pronged control measures are essential to mitigate the significant public health threat posed by this invasive vector.

## Data Availability

The datasets generated and/or analyzed during the current study are available from the corresponding author upon reasonable request. The newly generated sequences were deposited in the GenBank (database under accession numbers: PV017696, PV017745, PV017901, PV018825, PV018826, PV032622, PV032624, PV032625, PV032626, PV075239, PV256625, and PV256626.

## Abbreviations

CI: Cytoplasmic incompatibility
*COI*: Cytochrome* c* oxidase subunit I
DI–IV: Domains I–IV
F1534L: Leucine substitution at phenylalanine position F1534 of the VSSC
*ITS2*: Internal transcribed spacer 2
*kdr*: Knockdown resistance
mtDNA: Mitochondrial DNA
PYs: Pyrethroids
VSSC: Voltage-sensitive sodium channel protein
*vssc*: Voltage-sensitive sodium channel gene
*w*AlbA: *Wolbachia* strain (supergroup A) in *Ae. albopictus*
*w*AlbB: *Wolbachia* strain (supergroup B) in *Ae. albopictus*
*w*AlbA + *w*AlbB: superinfection of *w*AlbA and *w*AlbB strains in* Ae. albopictus*
*w*Mel: *Wolbachia* strain in *Drosophila melanogaster*
*wsp*: *Wolbachia* surface protein gene
